# LRRML: a conformational database and an XML description of leucine-rich repeats (LRRs)

**DOI:** 10.1186/1472-6807-8-47

**Published:** 2008-11-05

**Authors:** Tiandi Wei, Jing Gong, Ferdinand Jamitzky, Wolfgang M Heckl, Robert W Stark, Shaila C Rössle

**Affiliations:** 1 Department of Earth and Environmental Sciences, Ludwig-Maximilians-Universität München, Theresienstr, 41, 80333 Munich, Germany; 2 Leibniz Supercomputing Centre, 85748 Garching, Germany; 3 Deutsches Museum, 80538 Munich, Germany

## Abstract

**Background:**

Leucine-rich repeats (LRRs) are present in more than 6000 proteins. They are found in organisms ranging from viruses to eukaryotes and play an important role in protein-ligand interactions. To date, more than one hundred crystal structures of LRR containing proteins have been determined. This knowledge has increased our ability to use the crystal structures as templates to model LRR proteins with unknown structures. Since the individual three-dimensional LRR structures are not directly available from the established databases and since there are only a few detailed annotations for them, a conformational LRR database useful for homology modeling of LRR proteins is desirable.

**Description:**

We developed LRRML, a conformational database and an extensible markup language (XML) description of LRRs. The release 0.2 contains 1261 individual LRR structures, which were identified from 112 PDB structures and annotated manually. An XML structure was defined to exchange and store the LRRs. LRRML provides a source for homology modeling and structural analysis of LRR proteins. In order to demonstrate the capabilities of the database we modeled the mouse Toll-like receptor 3 (TLR3) by multiple templates homology modeling and compared the result with the crystal structure.

**Conclusion:**

LRRML is an information source for investigators involved in both theoretical and applied research on LRR proteins. It is available at .

## Background

Leucine-rich repeats (LRRs) are arrays of 20 to 30 amino acid long protein segments that are unusually rich in the hydrophobic amino acid leucine. They are present in more than 6000 proteins in different organisms ranging from viruses to eukaryotes [1]. The structure of the LRRs and their arrangement in repetitive stretches of variable length generate a versatile and highly evolvable framework for the binding of manifold proteins and non-protein ligands [2]. The crystal structure of the ribonuclease inhibitor (RI) yielded the first insight into the three-dimensional molecular basis of LRRs [3]. It has a horseshoe shaped solenoid structure with parallel β-sheet lining the inner circumference and α-helices flanking its outer circumference. To date, there are over one hundred crystal structures available. All known LRR domains adopt an arc or horseshoe shape [1].

The LRR sequences can be divided into a highly conserved segment (HCS) and a variable segment (VS). The highly conserved segment consists of an 11 or 12 residue stretch with the consensus sequence LxxLxLxxN(Cx)xL. Here, the letter L stands for Leu, Ile, Val or Phe forming the hydrophobic core, N stands for Asn, Thr, Ser or Cys, and x is any amino acid. The variable segment is quite diverse in length and consensus sequence, accordingly eight classes of LRRs have been proposed [4,5]: 'RI-like (RI)', 'Cysteine-containing (CC)', 'Bacterial (S)', 'SDS22-like (SDS22)', 'Plant-specific (PS)', 'Typical (T)', 'Treponema pallidum (Tp)' and 'CD42b-like (CD42b)'.

The discrepancy between the numbers of structure-known LRR proteins and the structure-unknown ones triggered studies focusing on the homology modeling of LRR proteins [6-8]. Homology modeling is a computational method, which is widely used to identify structural features defining molecular interactions [8-10]. The modeling results are an important input for the design of biochemical experiments. The first step of homology modeling is the selection of a structure-known protein, which serves as a template for the unknown target structure. In practice, however, it is difficult to find a complete template which has a high enough sequence identity to the target repetitive protein (single template modeling), due to different repeat numbers and varying arrangements. This limitation can be overcome by combining multiple templates. First, the most similar structure-known LRRs are found for each LRR in the target sequence as a local template. Second, all local templates are combined to generate the multiple sequence alignments for the entire target sequence. Thus, it is possible to construct a start model for further investigation, even if no adequate single template is available. Such an approach, however, requires a comprehensive database of LRRs to extract adequate template candidates. So far, the individual three-dimensional LRR structures are not directly available from the established databases and there are only a few detailed annotations for them. Additional information such as sequence insertions and types is missing. In order to consolidate this information and to provide a source for homology modeling and structural analysis of LRR proteins, we developed LRRML, a database and an extensible markup language (XML) description of LRR structures.

## Construction and content

Structure-known LRR proteins were extracted from the Protein Data Bank (PDB) [11] release Sept 10, 2008. In order to ensure that all LRR proteins were found, we combined three groups of search results. First, 'leucine rich repeat', 'leucine rich repeats', 'leucine-rich repeat', 'leucine-rich repeats', 'lrr' and 'lrrs' were used as key words in the PDB quick search; second, 'SCOP classification -> Alpha and beta proteins (a/b) -> Leucine-rich repeat' was used as options in PDB advanced search; third, 'CATH classification -> Alpha Beta -> Alpha-Beta Horseshoe -> Leucine-rich repeat' was used as options in PDB advanced search. Because of the irregularity (mutations and insertions in the sequence) of LRRs reliable identifications of LRRs contained in the LRR proteins could only be performed manually. We inspected the three-dimensional structures of the LRR proteins using molecular viewers and identified each LRR based on two criteria:

1. A LRR begins at the beginning of the highly conserved segment (HCS) and ends at the end of the variable segment (VS) (just before the HCS of the next LRR).

2. The HCS of a LRR must pose a typical conformation, i.e. a short β-sheet begins at about position 3 and a hydrophobic core is formed by the four L residues at position 1, 4, 6, and 11.

The LRRs were then manually classified according to the consensus sequences [4,5]. In addition to the eight canonical LRR classes listed in the background section we included a new class 'other' for the N-/C-terminal LRRs and some hyper-irregular LRRs. Table 1 illustrates the consensus sequences of the eight canonical LRR classes.

**Table 1:** Consensus sequences of the eight canonical LRR classes [4,5].

Classes	HCS	VS
Typical type (T)	LxxLxLxxNxL	xxLxxxxLxxLxx
Bacterial type (S)	LxxLxLxxNxL	xxLPx(x)LPxx
Ribonuclease inhibitor-like type (RI)	LxxLxLxxNxL	xxxxxxxLxxxLxxxxx
SDS22-like type (SDS22)	LxxLxLxxNxL	xxLxxLxxLxx
Cysteine-containing type (CC)	LxxLxLxxCxxL	TDxxxxxLxxxCxx
Plant-specific type (PS)	LxxLxLxxNxL	xxxLPxxLGxLxx
Treponema pallidum type (Tp)	LxxLxLPxxLxx	LxxxAFxxCxx
CD42b type (CD42b)	LxxLxLxxNxL	xxLPxxxxxxxxx

L: Leu, Ile, Val, Phe; N: Asn, Thr, Ser, Cys; P: Pro; T: Thr; D: Asp; G: Gly; A: Ala; F: Phe; C: Cys; x: random residues.

During the LRR identification and classification all sequence insertions longer than 3 residues were annotated. About one tenth of entries have insertions longer than 3 residues while few entries have deletions, which suggests that the evolution of LRRs may prefer insertion to deletion.

**Figure 1:**
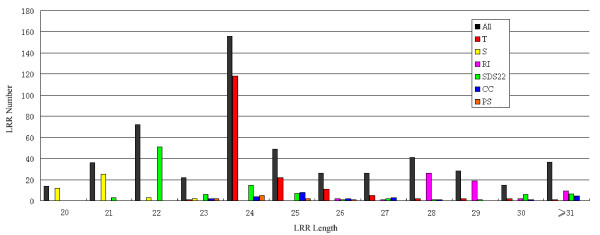
**LRR entry length distribution**. The most common entry lengths vary from 20 to 29. Each LRR class has a characteristic length distribution. Some entries have a sequence length larger than 29 due to insertions.

The LRRML release 0.2 contains 1261 LRR entries from 112 PDB structures. Among them 548 LRRs are distinct on sequence level, indicating that different molecules can share identical LRRs. By superimposition, we found that they also have highly similar structures. This fact enhances the confidence in modeling LRR proteins using multiple LRR templates. A histogram of entry length distribution (Figure 1) shows that the LRR lengths are concentrated in the interval from 20 to 29, which covers the characteristic lengths of consensus sequences of the eight canonical LRR classes. Some entries have a sequence longer than 30, because they contain large insertions. Table 2 presents the distribution of LRR entries and PDB entries over the nine classes respectively. The classification results are consistent with a previous report which showed that LRRs from different classes never occur simultaneously in the same protein and have most probably evolved independently [4]. Exceptions to this rule are the T and S types which often exist in the same protein forming the super motif 'STT' [12]. It is assumed that both evolved from a common precursor [1].

**Table 2:** Numbers of LRR and PDB entries (release 0.2) in the nine LRR classes.

	T	S	RI	SDS22	CC	PS	Tp	CD42b	Other	Total
LRR structures	272	72	151	372	184	10	0	0	200	1261

LRR entries	169	40	59	114	28	10	0	0	128	548

PDB entries	32		13	50	16	1	0	0	-	112

Up to present, no crystal structures for LRR proteins of Tp/CD42b types are determined. Different from other LRR types, the S type and T type LRRs evolved from a common precursor [1] and thus can exist in the same PDB entry simultaneously.

Currently, there are several protein databases containing information on LRRs, such as Pfam [13], InterPro [14], SMART [15] and Swiss-Prot [16]. These databases predict the LRR numbers and boundaries for their LRR protein entries by various computational methods, no matter whether the entries have known three-dimensional structures or not, thereby 'false negative' occurs frequently. Table 3 lists the numbers of structure-known LRR proteins and their LRRs covered by these databases. As more detailed examples, LRR numbers of LRR proteins from different classes reported by the established databases are compared in Table 4. Additionally, the individual three-dimensional LRR structures are not directly available from these databases. In order to combine the information required for homology modeling and structural analysis, LRRML is provided with three prominent characteristics:

**Table 3:** Coverage of LRR proteins with PDB structures of different databases.

Databases	Numbers of LRR proteins with PDB structures	Numbers of identified LRRs
InterPro	62	325
Swiss-Prot	98	997
Pfam	48	173
SMART	84	547
LRRML	112	1261

The results were obtained on October 13, 2008.

**Table 4:** Comparison of LRR numbers of different LRR proteins by different databases.

PDB codes	Protein functions	LRR classes	InterPro	Swiss-Prot	Pfam	SMART	LRRML
2A0Z	Immune System	T	18	22	7	20	25
1G9U	Toxin	S	7	15	1	0	15
2FT3	Structural Protein	T+S	8	8	5	9	12
1K5D	Signaling Protein	RI	2	8	0	0	11
1GWB	Glycoprotein	SDS22	6	6	4	7	8
2P1M	Signaling Protein	CC	2	16	0	6	18
1OGQ	Inhibitor	PS	7	10	2	0	10

All listed LRR numbers include N-/C-terminal LRRs. To date, only the LRRML database contains the complete set of LRRs of all LRR proteins with known structures. The results were obtained on October 13, 2008.

1. Each database entry is an individual three-dimensional LRR structure, which was identified with high accuracy.

2. Extensive annotations, such as systematic classification, secondary structures, HCS/VS partitions and sequence insertion, are provided.

3. LRRs were extracted from all structure-known LRR protein structures from PDB.

## XML description

The extensible markup language (XML) was standardized in the 90s and is well established as a format for hierarchical data. It can be queried and parsed more easily by application programs. Therefore, more and more biological databases use the XML as data saving format and database management system (DBMS) [17-19]. LRRML was designed by using eXist [20], an XML DBMS, and using XPath/XQuery [21] for processing queries and web forms. We developed a LRR markup language (LRRML) for exchanging and storing LRR structures. It consists of four blocks of information:

1. The sequence information (XML tag <l:Sequence>): amino acid sequence and sequence length.

2. The classification information (XML tag <l:Type>): class name and consensus sequences.

3. The sequence partitions (XML tag <l:Regions>): amino acid sequence, position, length and insertion of HCS and VS.

4. The corresponding PDB sources (XML tag <l:Sources>): ID, chain, LRR number and classification of the source PDB entries; serial number, position, DSSP [22] secondary structure and three-dimensional coordinates of the current LRR in these source PDB entries.

**Figure 2:**
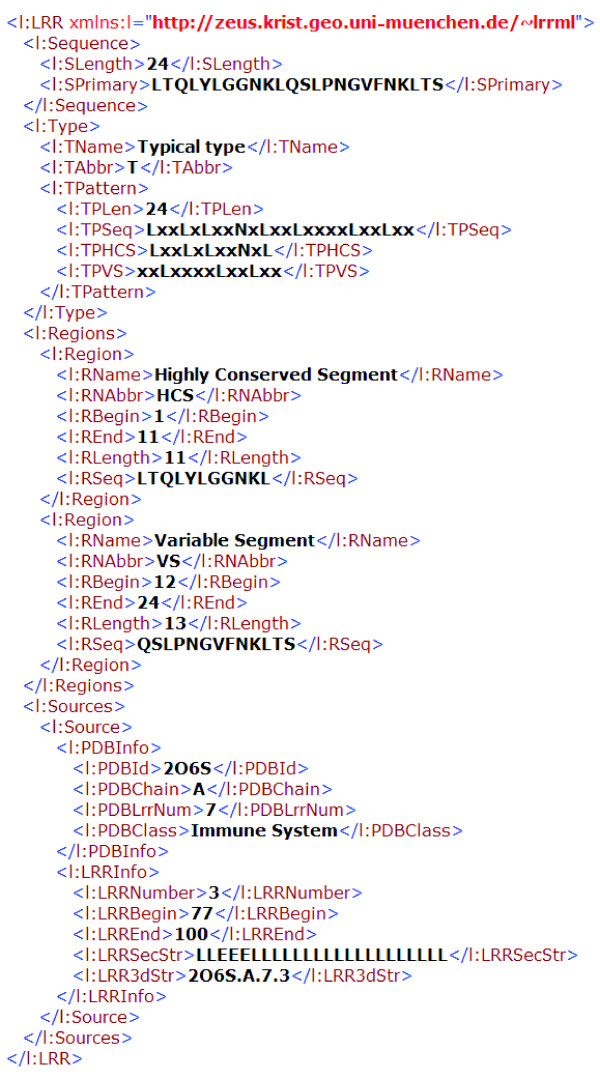
**The LRRML description of a LRR structure**. This entry is a 24 residue long typical LRR. The first 11 residues compose its HCS and the last 13 residues compose its VS (no insertions). It is contained only in the chain A of PDB structure 2O6S (a protein involved in the immune system). It is the third one of the 7 LRRs of 2O6S, from position 77 to 100. Its secondary structure was extracted from DSSP and its three-dimensional coordinate file is available though the hyperlink on the corresponding web page.

An example describing the LRR3 from PDB entry 2O6S is shown in Figure 2. The document type definition (DTD) file of LRRML is provided asAdditional file 1.

## Utility

### Web application

The entire database can be browsed by LRR IDs or by PDB IDs. When browsing, the entries appear in a summary table containing at first ID, type and sequence. Clicking on an ID opens an XML Stylesheet (XSLT) [21] converted HTML web page that presents the entry in detail. The original XML file and the coordinates file in PDB format can also be downloaded. The XSLT file used is provided as Additional file 2. Aside from the textual view, a LRR structure can be visualized by the online molecular viewer Jmol [23]. After loading, users can change the view settings flexibly by themselves. LRRML is provided with various search functions, including PDB ID search which returns all LRRs contained in this PDB structure, class search which returns all LRRs of this class, or length search which returns all LRRs with this sequence length. To simplify the homology modeling, the similarity search was implemented. It returns the structures of the most similar LRRs for a structure-unknown LRR. The target LRR sequence can be searched against the entire database, a certain LRR class or LRRs with a certain length. At first, a global pair wise sequence alignment with sequence identity will be generated for the target LRR and each of the LRRs in the user selected set. Then, the most similar LRRs will be returned as template candidates, ranked by sequence identity.

The DBMS provides a REST-style application programming interface (API) through HTTP, which supports GET and POST requests. A unique resource identifier (URI) '...' is treated by the server as path to a database collection. Also, request parameters can help select any required elements. For example, '_query' executes a specified XPath/XQuery; the URL "" returns all the S type LRRs.

### Application in homology modeling

LRRML was designed as a tool for template selection in homology modeling of LRR proteins. Traditionally, the template used in homology modeling is one or more full length protein structures obtained via similarity search. Nevertheless, due to the different repeat numbers and arrangements of LRRs, the sequence identity between the target and the full length template is usually not high enough for homology modeling. With LRRML the most similar structure-known LRR can be found for each LRR in the target sequence as a local template. The combination of all local templates through multiple alignments helps to achieve a high sequence identity to the target.

**Figure 3:**
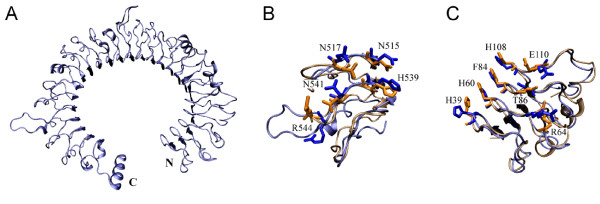
**Comparison of model and crystal structure of mouse TLR3 ectodomain at the two ligand interaction regions**. Blue: structure obtained by homology modeling; orange: crystal structure (PDB code: 3CIG). (A) The modeled backbone structure of mouse TLR3 ectodomain. (B) Model and crystal structure superimposed at the N-terminal interaction region. The root mean square deviation is 1.96 Å. (C) Superimposition at the C-terminal interaction region. The root mean square deviation is 1.9 Å. The reported interacting residues are presented with side chain and labelled with residue name and position in (B) and (C).

**Figure 4:**
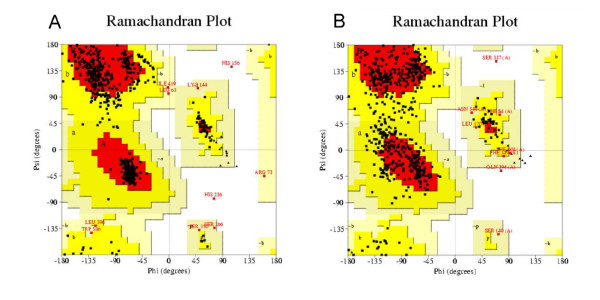
**Ramachandran plot of model and crystal structure of mouse TLR3 ectodomain**. (A) Predicted model of mouse TLR3 ectodomain. (B) Crystal structure of mouse TLR3 ectodomain. The different colored areas indicate 'disallowed' (white), 'generously allowed' (light yellow), 'additional allowed' (yellow), and 'most favored' (red) regions.

As test case we modeled the structure of mouse Toll-like receptor 3 (TLR3) ectodomain. We assumed that the structure of mouse TLR3 ectodomain were unknown and excluded the LRRs of mouse/human TLR3 ectodomain from LRRML. Through similarity search the optimal template for each of the 25 LRRs in mouse TLR3 was found. The sequence identity between each LRR pair (target/template LRR) is listed in Table 5. Then a 26-line multiple alignment was generated by the 25 template sequences and the target sequence as the input of MODELLER 9v3 [24]. The resulting three-dimensional model (Figure 3A) was evaluated by PROCHECK [25], with 98.2% residues falling into the most favored or allowed regions of the main chain torsion angles distribution, whereas the result of the TLR3 crystal structure (PDB code: 3CIG) was 98.6% (Figure 4). The mouse TLR3 has been shown to bind double-stranded RNA ligand with both N-terminal and C-terminal sites on the lateral side of the convex surface of TLR3 [26]. The N-terminal interaction site is composed of LRRNT and LRR1-3, and the C-terminal site is composed of LRR19-21. We superimposed the resulting model onto the crystal structure of mouse TLR3 ectodomain at the two interaction sites by using SuperPose v1.0 [27]. The root mean square deviations of the structures are 1.96 Å and 1.9 Å respectively (Figure 3B/C), indicating that the predicted model sufficiently well matched the crystal structure and was useful for prediction of ligand interaction sites. These results demonstrate that homology modeling using combined multiple templates obtained from LRRML can create valuable information to trigger further biochemical research. Interpretation of structural details, however, should be done exercising due care.

**Table 5:** Sequence identities (%) of target-template LRR pairs.

	NT	1	2	3	4	5	6	7	8	9	10	11	12	13	14	15	16	17	18	19	20	21	22	23	CT	Avg
LRRML ID	406	65	212	465	151	177	110	259	8	203	64	293	270	357	65	152	259	316	152	239	239	92	80	101	173	---
PDB source	1XWD	2O6S	1G9U	1OZN	1XKU	1SQ0	2FT3	2V9S	1IO0	1H6U	2O6S	2Z64	2Z62	2Z81	2O6S	1XKU	2V9S	2Z7X	1XKU	1P8V	1P8V	2ID5	2O6Q	2ID5	1W8A	---
Identity (%)	47.60	45.83	45.83	41.67	50.00	50.00	46.15	41.67	41.38	42.31	50.00	50.00	33.33	41.67	42.86	50.00	40.00	37.50	38.46	50.00	45.83	50.00	41.67	40.00	39.29	44.12

In the header line, 1–25 denote canonical LRRs; NT and CT denote N-/C-terminal LRRs.

## Conclusion

A specialised conformational leucine-rich repeats database called LRRML has been developed. It is supported by an XML database management system and can be searched and browsed with either an easy-to-use web interface or REST like interface. The interface is suitable for most graphical web browsers and has been tested on the Windows, Mac and Linux operating systems. LRRML contains individual three-dimensional LRR structures with manual structural annotations. It presents useful sources for homology modeling and structural analysis of LRR proteins. Since the amount of structure-determined LRR proteins constantly increases, we plan to update LRRML every 2 to 3 months.

## Availability and requirements

This database is freely available at .

## Authors' contributions

TW and JG drafted the manuscript, extracted the data, compiled the database, wrote the code for the web interface and performed the statistical analysis. FJ, WMH, RWS and SCR conceived of the study, built the database server, participated in the database design and coordination and helped to draft the manuscript. TW and JG should be regarded as joint first authors. All authors read and approved the final manuscript.

## Supplementary Material

Additional file 1 The document type definition (DTD) file of LRRML. Click here for file

Additional file 2 The XML Stylesheet (XSLT) of LRRML. Click here for file
